# Supplementary material for: Psycho-social factors associated with type two diabetes remission through lifestyle intervention: A scoping review
Source: PLoS One. 2023 Nov 16;18(11):e0294344. doi: 10.1371/journal.pone.0294344 (PMC10653481; doi:10.1371/journal.pone.0294344)
Supplement: S2 Table — (DOCX) [file pone.0294344.s002.docx]

**S2 table: Search terms used for the database searches of EMBASE, MEDLINE, CINAHL ultimate, PsychINFO and PsycArticles.**

| **Population**  Terms associated with diabetes | "type two diabetes"  "Diabetes Mellitus, Type 2"  "type two diabetes mellitus"  “T2D”  “T2DM” |
| --- | --- |
| Terms associated with remission | "remission"  "Disease Remission"  revers*  "cure" |
| **Concept**  Terms associated with Psycho-social factors | diet and nutrition  "Diet”  weight loss or weight reduction or lose weight or obesity or overweight or weight management  glycemic  glucose monitoring or glucose control or glycemic control or sugar control  support  "Support, Psychosocial"  psychosocial factors or psychosocial impacts or psychosocial effects  "Psychosocial Aspects of Illness  "Psychosocial Intervention"  depression  diabetes distress or diabetes related distress or diabetes-specific emotional distress  physical activity or exercise or fitness or physical exercise  behavioural change  "hope"  motivation*  self-efficacy or confidence or self esteem |
