# Supplementary material for: Psycho-social factors associated with type two diabetes remission through lifestyle intervention: A scoping review
Source: PLoS One. 2023 Nov 16;18(11):e0294344. doi: 10.1371/journal.pone.0294344 (PMC10653481; doi:10.1371/journal.pone.0294344)
Supplement: S3 Table — (DOCX) [file pone.0294344.s003.docx]

**S3 table: Results from database search of CINAHL Ultimate (conducted 26/9/22).**

| # | Keyword/s or Search term/s |  |
| --- | --- | --- |
| 1 | "type two diabetes" | 55,787 |
| 2 | (MM "Diabetes Mellitus, Type 2") | 55,669 |
| 3 | "type two diabetes mellitus" | 53 |
| 4 | “T2D” | 4505 |
| 5 | “T2DM” | 7965 |
| 6 | #1 OR #2 OR #3 OR #4 OR #5 | 59,286 |
| 7 | "remission" | 27,625 |
| 8 | (MM "Disease Remission") | 1903 |
| 9 | revers* | 80,907 |
| 10 | "cure" | 20,212 |
| 11 | #7 OR #8 OR #9 OR #10 | 127,052 |
| 12 | (#7 OR #8 OR #9 OR #10) AND (#6 AND #11) | 1468 |
| 13 | diet and nutrition | 36,069 |
| 14 | (MH "Diet+") | 140,112 |
| 15 | weight loss or weight reduction or lose weight or obesity or overweight or weight management | 187,941 |
| 16 | glyc#emic | 32,894 |
| 17 | glucose monitoring or glucose control or glycemic control or sugar control | 38,341 |
| 18 | support | 537,640 |
| 19 | (MH "Support, Psychosocial+") | 100,160 |
| 20 | psychosocial factors or psychosocial impacts or psychosocial effects | 528,392 |
| 21 | (MH "Psychosocial Aspects of Illness+") OR (MM "Psychosocial Intervention") | 223,230 |
| 22 | depression | 202,605 |
| 23 | diabetes distress or diabetes related distress or diabetes-specific emotional distress | 1097 |
| 24 | physical activity or exercise or fitness or physical exercise | 293,241 |
| 25 | behavioural change | 19,685 |
| 26 | (MM "Behavioral Changes") | 5,160 |
| 27 | "hope" | 29,608 |
| 28 | motivation* | 71,804 |
| 29 | self-efficacy or self efficacy or confidence or self esteem | 477,875 |
| 30 | #13 OR #14 OR #15 OR #16 OR #17 OR #18 OR #19 OR #20 OR #21 OR #22 OR #23 OR #24 OR #25 OR #26 OR #27 OR #28 OR #29 | 2,052,473 |
| 31 | #12 AND #30 | 969 |
| 32 | LIMIT: 2009 | 836 |
| 33 | LIMIT: English Language | 829 |
