# Supplementary material for: Psycho-social factors associated with type two diabetes remission through lifestyle intervention: A scoping review
Source: PLoS One. 2023 Nov 16;18(11):e0294344. doi: 10.1371/journal.pone.0294344 (PMC10653481; doi:10.1371/journal.pone.0294344)
Supplement: S4 Table — (DOCX) [file pone.0294344.s004.docx]

**S4 table: Data extraction form one- detailing study, country, participants, design, lifestyle intervention, number of psycho-social scales used and the effect of these on remission outcomes.**

| No. | Article | Country | Design | Participants | Lifestyle intervention | Psycho-social scales | Effect on psycho-social factors on remission | Notes |
| --- | --- | --- | --- | --- | --- | --- | --- | --- |
| 1 | Ades et al (2015) | Canada | Single arm | N=12, overweight/obese (BMI 35.8±4.3 kg/m2), and recently (< 1 year) diagnosed with T2DM. | 6-month exercise and weight loss program.  1-3, 45–60-minute sessions/week of supervised on-site exercise. Caloric goal was 500 kcal less than predicted maintenance calories | None | N/A |  |
| 2 | Athinarayanan et al (2019) | USA | Non-randomised clinical trial | N=262 with T2D placed in continuous care intervention (CCI), N=87 with T2D in usual care group (UC) | CCI group: Nutritional ketosis through carbohydrate restriction. | None | N/A |  |
| 3 | Bhatt et al (2017) | India | Single arm | N=12 with T2D | Low calorie diet (~1000 kcal/day) through meal replacement protein formula. Home blood glucose monitoring | None | N/A |  |
| 4 | Bynoe et al (2020) | Barbados | Single arm | N=25, with T2D diagnosed within past 6 years, not on insulin, BMI ≥ 27 kg/m2 | 8-week liquid (760 calorie) diet, then 4 week supported transition to solid food. | None- however qualitative element | Rigidity of liquid diet removed need to make food choices. Difficulty in transition to solid food. Food environment, peer pressure and cultural expectations created difficulties. Gender differences in perception of self during rapid weight loss. Main facilitator was social support. |  |
| 5 | Chen et al (2022) | USA | Retrospective cohort study | N=9,900 adults with T2D in medically underserved area | Patient self-management- no information on method chosen to achieve weight loss | None | N/A |  |
| 6 | Correa et al (2022) | Brazil | Case study | N=1, 35 year old male, T2D for 5 years | Very low-carbohydrate ketogenic diet (VLCKD) through commercial multidisciplinary weight-loss program (PnK® method) | None | N/A |  |
| 7 | Cox et al (2019) | USA | Case study | N=65 year old female. T2D for 26 years | Clinically prescribed  ketogenic diet. Time restricted eating. Guided HIIT (high intensity  interval training) | The Patient Health Questionnaire 9 (PHQ-9), General Self Efficacy scale (GSE) | PHQ-9 score was substantially decreased from 17 (moderately severe depression) to 0 (minimal depression). GSE also improved from 20 to 39, reflecting increased confidence in the patient's self-management of diabetes. Patient self-reported heightened self-confidence, increased self-efficacy, increased energy, improved sleep and stability in her mood with clearer cognition. |  |
| 8 | Dambha-Miller et al (2021) | UK | Retrospective Cohort Study | N=56,120 | Patient self-management- no information on method chosen to achieve weight loss | None | N/A |  |
| 9 | Dave et al (2019) | India | Single arm | N=45 | Intensive lifestyle intervention based on ADA dietary guidelines. Personalized, individualized and tailored meal plans. Bespoke exercise schedule. Self-monitoring of blood glucose. | None | N/A |  |
| 10 | De Hoogh et al(2021) | Netherlands | Single arm | N=15 | Diabetes subtyping  (“diabetyping”) lifestyle intervention (DLI). Very  low-calorie diet for only one week, followed by a six-week low-carb diet and then gradual reintroduction to a Mediterranean diet.  Self-monitoring by glucose meter. When necessary dietary interventions were intensified by exercise and intermittent fasting | None | N/A |  |
| 11 | Esposito et al (2014) | Italy | Follow up to RCT | N= 215 men and women with  newly diagnosed T2D | 2-armed study participants randomised to low-carbohydrate  Mediterranean diet (LCMD; n = 108) or a low-fat diet (n = 107). Tailored programme of physical activity | None | N/A |  |
| 12 | Gregg et al (2012) | USA | Follow up to RCT | N= 4503 with T2D. BMI ≥25 | Patients randomised to intensive lifestyle intervention (ILI) involved calorie reduction through  liquid  meal replacements and increased physical exercise. | None | N/A |  |
| 13 | Karter et al (2014). | USA | Retrospective cohort study | N= 122,781 adults with T2D | Patient self-management- no information on method chosen to achieve weight loss | None | N/A |  |
| 14 | Lagger et al (2018). | Switzerland | Single arm | N= 17 with recently diagnosed T2D, not on insulin | Therapeutic education programme delivered as 5 day outpatient programme. Followed by 12 months of 1hr sessions. No specific dietary or exercise requirements discussed. | Hospital Anxiety and Depression scale (HAD) given to the patient. | HAD anxiety scores decreased: pre 7.8 (6.6) and post 5.7 (4.0) but HAD depression increased: pre 8.9 (5.8), post 9.2 (4.2). | No differentiation between those who achieved remission and those who did not so can only interpret this as a response to the intervention not achieving remission.  Additional questions posed in interview |
| 15 | Lean et al (2018). | UK | Open-label, cluster-randomised trial  (DiRECT) | N=306 | Counterweight-Plus weight management  Programme -total diet  replacement phase using a low energy formula diet  (825–853 kcal p/day)  for 3 months, structured  food reintroduction of 2–8 weeks. Step counters and physical  activity strategies introduced in second stage. | Quality of life- EQ-5D visual analogue scale | Q of L increased from 66.4 (19.2) at baseline to 73.7 (19.0) at 12 months in the intervention group. In the control group Q of L decreased from 72.0 (16.9) at baseline to 69.1 (15.6) after 12 months. | More remission in intervention than control.  Diabetes remission was achieved in 68 (46%) participants in the intervention group and  six (4%) participants in the control group so more confidence that PS factors linked to remission. |
| 16 | Lean et al (2019). | UK | Follow up study to randomised trial  (DiRECT) | N=256 | As above -2 year follow up | Quality of life- EQ-5D visual analogue scale.  EQ5D Health Utility Score | After 24 months Q of L in the intervention group had increased from 66.4 (19.2) at baseline to 75.2 (17.3) an 8.2 (20.1) change. In the control group the baseline measure 72.1 (19·6) had decreased at 12 months 69·1(15·6) but by 24 months had increased to 74·0 (16·8)-a 1·7 (15·1) change over the 24-month period.  EQ5D health utility score (unreported in 2018 study) changed from 0.798 at baseline in the intervention group to 0.819 at 24 months. In the control group this changed from 0.802 at baseline to 0.788 at 24 months. | At 24 months, 53/149 (35·6%) of those commencing the intervention and 5/149 (3·4%) in  the control group had remission. Number who have sustained remission dropped so less confidence that PS factors associated with remission. |
| 17 | Marples et al (2022) | UK | Service evaluation | N =37 (but only 29 completed intervention) | Low energy diet using Counterweight TDR products (825–853 kcal/day) for 12 weeks, 9 week food reintroduction stage. where increase in physical activity was encouraged  with the aim of at least 150 min of moderate  activity a week, then 31 week weight maintenance stage. | Diabetes Distress Scale (DDS),  Patient Health Questionnaire (PHQ9),  Generalised Anxiety Disorder (GAD-7),  Work and Social Adjustment Scale (WAS),  Rosenberg self-esteem scale and Binge  Eating Scale (BES). | DDS- reduced from 2.3 (1.0) at baseline to 1.8 (0.9) at 12 months.  PHQ9- reduced from 5.3 (4.8) at baseline to 3.4 (4.4) at 12 months  GAD-7 3.7 (4.4) at baseline reduced to 3.4 (4.7) at 12 months.  WAS 6.3 (6.7) at baseline increased to 6.5 (9.5) at 12 months.  Rosenberg S.E 21.3 (5.2) at baseline increased to 24.1 (5.0) at 12 months.  BES 9.3 (6.0) at baseline reduced to 7.7 (5.7) at 12 months. | No differentiation between those who achieved remission and those who did not so can only interpret this as a response to the intervention not achieving remission. |
| 18 | Mottalib et al (2015) | USA | Single arm | N=126 T2D patients (BMI ≥ 30 kg/m2). Analysis is based on completers after one year n= 88 | Why WAIT (Weight Achievement and Intensive Treatment) program. Medication adjustment. Blood glucose monitoring. Dietary evaluation calories reduced by approximately 500 cals a day from usual consumption. Partial meal replacement (breakfast and lunch) using BOOST Glucose Control. Individualised, graded exercise plan. | None | N/A |  |
| 19 | Oser et al (2022) | USA | Single arm | N=17 adults recently diagnosed with T2D | Glycemic excursion minimization (GEM) which is an empowerment programme. Participants were given a CGM reader, sensor supplies, and  a Fitbit Charge 3 activity monitor. 5 days was spent tracking glucose excursions, followed by 14 days reducing/eliminating carbohydrates and then a further period of self-monitoring. Intervention was primarily psycho-social. | Diabetes knowledge, Diabetes Empowerment, Diabetes Distress (emotional subscale), Diabetes  Distress (regimen subscale),  and depressive symptoms. | Diabetes knowledge increased from 15.5 (3.0) pre-treatment to 15.9 (3.0) at 3 months post GEM.  Diabetes Empowerment increased from  31.0 (5.9) 34.6 (3.8).  Diabetes distress (emotional) reduced from 2.2 (0.8) to 1.8 (1.0).  Diabetes distress (regimen) reduced from 2.8 (1.4) to 1.8 (0.9)  Depressive symptoms reduced from 6.1 (4.5) to 2.3 (3.9) | No differentiation between those who achieved remission and those who did not so can only interpret this as a response to the intervention not achieving remission. |
| 20 | Rehackova et al (2017). | UK | Qualitative | N=18 | Study participants had been involved in the Counterbalance study, comprising  an 8-week VLED, followed by a 2-week stepped return to an  isocaloric diet of usual foodstuffs. | None- qualitative | Motivation- linked to body image, desire to improve health and quality of life, to reduce burden of diabetes management and medication.  Barriers to adherence (and therefore potential remission), availability of social support, triggers of physical and social  environments, availability  of self-regulatory skills.  Feelings of sadness, loneliness or stressful  experiences affected adherence.  Facilitators: ease of VLED and satiety, rapid results, social support and involvement in a research study, positive reinforcement from change in clothing size, compliments from others, peer support from others on weight loss journeys, ability to use distraction and avoidance for behaviour regulation. Receiving biofeedback was identified as important. | Group responses analysed as whole- no differentiation between those who achieved remission and those who did not. |
| 21 | Rehackova et al (2020). | UK | Qualitative (longitudinal) | N=11 | Study participants had been involved in the Counterbalance study, comprising  an 8-week VLED, followed by a 2-week stepped return to an  isocaloric diet of usual foodstuffs. | None-qualitative | Key themes included: behavioural interdependence (difficulty making health behaviour decisions different from the behaviours of others in their social environment) shifting to behavioural autonomy. Behavioural contagion (changes in other people’s health behaviours due to participant’s behaviour change). Difficulty in decision making and autonomy in transition from rigid eating to flexible restraint. Shift in self-identity. |  |
| 22 | Rehackova et al (2022). | UK | Qualitative | N=34 | Study participants had been involved in the DiRECT study. Total diet replacement for 12 weeks (extendable  up to 20 weeks at the request of the participant),  followed by structured food reintroduction (2–8 weeks) and weight loss management for up to 2 years. | None-qualitative | Expectations: motivation to change, trust in study, prospect  of diabetes remission, medication reduction, and weight loss.  Process: Learning new behaviours and overcoming difficulties with adherence to TDR.  Ability to adapt to the regime change by using the available behavioural support and strategies.  Rapid improvements in  physical and psychological well-being motivating factor.  Transition to regular food as  disruption of adaptation. Learning maintenance behaviours and overcoming difficulties with WLM.  Using meal replacements to  facilitate WLM.  Increased awareness of eating behaviours and continuous WLM effort.  The roles of behavioural and social support . | Population weighted in favour of those who had achieved remission. |
| 23 | Rein et al (2022). | Israel | Randomised crossover trial | N=23 newly diagnosed and naïve to glucose lowering medication in trial 16/23 in the following intervention | Crossover trial 2 weeks PPT diet (uses algorithms that  integrate clinical and microbiome features to predict personal postprandial glucose responses (PPGR), 1 week washout then 2 weeks Mediterranean style  (MED) diet or vice versa in the other group. PPT involved CGM. 16/23 took part in a 6 month PPT intervention. | None | N/A |  |
| 24 | Ried-Larsen et al (2019) | Denmark | Follow up to randomized clinical trial (U-TURN) | N=98 non-insulin dependent T2D (duration <10 years), | Standard care group involved medical counselling, education and lifestyle advice. U-TURN lifestyle intervention group: supervised resistance and aerobic exercise for 30-60 mins, 5/ 6 days per week.  Individually tailored  dietary plans with an  energy intake restriction during the initial 4 months. | None | N/A |  |
| 25 | Romano et al (2019). | Italy | Single arm | N=20 | Very low calorie ketogenic diet (VLCKD) with protein supplement and vegetables. Average caloric content  450–600 kcal/day for women and 650–800 kcal/day for men. 8 week intervention. | None | N/A |  |
| 26 | Sarathi et al (2017) | India | Single arm | N= 32 patients with newly diagnosed type 2 DM. | Intensive lifestyle therapy (ILT) including low‑calorie diet [1500 kcal/day] and brisk walking for 1 h/day. | None | N/A |  |
| 27 | Steven et al (2016). | UK | Single arm (Counterbalance) | N=30 | Counterbalance study: VLCD (liquid diet formula) for 8 weeks; a stepped return to  isocaloric intake of normal food over 2  weeks; and a structured, individualized  weight maintenance program over 6  months. | None | N/A |  |
| 28 | Taheri et al(2020) | Quatar | Open-label,  parallel-group, randomised controlled trial (DIADEM-I) | N= 158 enrolled. N=147 completed. Aged 18–50 years, with a short diabetes  duration (≤3 years), had a BMI of 27・0 kg/m2 or more, | Control group (N=77) usual care.  Intervention group (N=70) 12-week total  diet replacement phase, (800–820 kcal/day) using Cambridge Weight Plan followed by a 12-week structured food reintroduction  phase. Thereafter, participants managed their own energy restricted  food intake and lifestyle changes for 6 months. Physical activity support initially focused  on walking (with an aim of at least 10 000 steps per day),  followed by recommendation of increasing  unsupervised activity to at least 150 min/week. Participants  were provided with a wrist-worn accelerometer and  were directed to smartphone apps to monitor food intake  and activity | EuroQol 5  Dimensions (EQ-5D) visual analogue scale,  Impact of weight on quality of life-lite  (IWQoL-Lite)  Hospital Anxiety and Depression scale (HAD) | Q of L increased in the intervention group from 79.89 (9.36) at baseline to 83.81 (11.55) a change of 4.32 (16.80). In the control group Q of L decreased from 81.96 (15.11) to 80.98 (16.73) a change of –1.03 (16.51).  Between baseline and 12 months, IWQoL-Lite scores  were increased in the intervention group 12·3 (16·9) and in the control group 6·6 (13·7)  HAD: There was no difference between groups in terms of mental health  outcomes at 12 months. | Diabetes remission occurred in 43 (61%)  of 70 participants in the intervention group compared  with nine (12%) of 77 participants in the control. Normoglycaemia occurred in 23 (33%) of  70 participants in the intervention group and three (4%)  of 77 participants in the control group- so more confidence that the difference in psycho-social factors reflects remission. |
| 29 | Tangelloju et al (2019). | USA | Retrospective cohort study | N= 10,059 in non-bariatric Medicare patients 65 years and older. | Patient self-management- no information on method chosen to achieve weight loss | None | N/A |  |
| 30 | Thom et al (2021) | UK | Follow up to an RCT (DiRECT) | N=149 (intervention group in DiRECT) | Counterweight-Plus weight management  Programme -total diet  replacement phase using a low energy formula diet  (825–853 kcal p/day)  for 3 months, structured  food reintroduction of 2–8 weeks, ongoing structured  programme with monthly visits. Step counters and physical  activity strategies introduced in second stage. | Quality of life- EQ-5D visual analogue scale (VAS).  EQ5D Health Utility Score (all 5 dimensions analysed separately)  Binge Eating Score | Q of L VAS: No remission group (n=76) 62.9 (19.6). Achieved remission after 24 months (n=53) 73.1 (18.1).  EQ-5D health utility: no remission group: 0.7 (0.3) increased to 0.9 (0.2) in those who achieved remission after 24 months. For every subscale (mobility, self-care, activities, pain, anxiety and depression) the number and % of those experiencing problems was reduced in the 24-month remission group when compared to the non-remitters.  Binge eating: 1.4 (1.5) in no remission group, reduces to 1.2 (1.2) in remission at 24 months group. | Interestingly in group who achieved remission at 12 but not 24 months this EQ-5D VAS is 57.4 (13.8) against an average of all participants of 65.8 (19.1) Binge eating is also increased from the average 1.3 (1.3) to 1.5 (1.3) in those in remission at 12-months but not 24. These factors may give some indication of why they were unable to sustain remission. |
| 31 | Umphonsathien et al (2019). | Thailand | Single arm | N=19 completed. (HbA1C) ≥6.5% (48 mmol/  mol), DM duration <10 years, BMI 23–30 kg/m2 | Initially participants were tried  on a very low calorie diet (VLCD) (600 kcal/day for 10 days  to assess for compliance. Dietary record  and urine ketone were used to monitor their compliance. Those  who passed 90% rate of compliance were invited to continue on the caloric restriction period, during which participants received  a VLCD for 8 weeks (weeks 0–8). Then  transition period  for another 4 weeks (weeks 8–12). During this period, participants  received higher caloric intake in a stepwise fashion (800 kcal/day  [3,349 KJ/day] on week 9, 1,000 kcal/day [4,187 KJ/day] on week  10, 1,200 kcal/day [5,024 KJ/day] on week 11, and 1,500 kcal/day  [6,280 KJ/day] on week 12). During all three study periods, patients  were asked to monitor blood glucose levels by fingerstick at least  twice weekly and when necessary if hypoglycemia was suspected | Quality of life (QOL) assessed using an SF‐36 questionnaire  at weeks - 2, 4, 8, and 12. | Q of L improved throughout the intervention period.  Week -2 = 2,494 ± 161,  Week 4 2,695 ± 134  Week 8= 2,880 ± 111  Week 12 =3,040 ± 100 | Use of compliance period.  An intention‐to‐treat (ITT) analysis showed  that diabetes remission was achieved in 75% at both 8 and 12 weeks. |
| 32 | Umphonsathien, et al (2022). | Thailand | RCT | N=40 30-60 years, diagnosis with T2D within  the previous 10 years. BMI  ≥23 kg/m2 and HbA1C level  between 6.5 and 10%. | Participants randomised to 3 groups: 2 days per week VLCD,  4 days per week VLCD and the control group.  In the 2-week run-in period, participants were tried on  VLCD (total calorie intake of 600 kcal/day) for 10 days to  assess compliance. In the 18-week intermittent caloric restriction  period, participants received 2 or 4 non-consecutive days/  week of intermittent VLCD. Ad libitum food consumption was  allowed on non-restricted days. | Quality of life (QOL) assessed using an SF‐36 questionnaire | Baseline Q of L Control = 2,563 (163) 2-day VLCD =2,444 (151), ), 4 day VLCD =2,081 (151)  Week 10. Q of L control= 2,730 (116) 2-day VLCD = 2,785 (107), 4-day VLCD= 2,866 (107).  Week 20 Q of L control= 2,684 (127), 2-day VLCD= 2,757 (118), 4 day VLCD = 2,697 (118).  There was a significant improvement in quality of life scores in both intervention groups at week 10 and only in the 4 days/week at week 20. | At the end of week 20, diabetes remission was achieved in  29% of participants in  both VLCDs  groups compared with none of the participants in the control  group. So can only interpret this as a response to the intervention not achieving remission. |
| 33 | Unwin et al (2020) | UK | Service evaluation | Analysis of data on N=128 (27%) of the practice with T2D who opted to follow a low carbohydrate diet | For patients who opted to try a lower carbohydrate diet,  dietary advice was given as part of routine GP or practice  nurse consultations. Several educational resources were produced to  support patients and staff including a lower carbohydrate diet sheet and infographics. | None | N/A |  |
| 34 | Webster et al (2019). | South Africa | Service evaluation | N=28 completed the study. 24/28 assessed for a second time after 15 months | Participants self-managed a low  carbohydrate high fat (LCHF) diet. | None but qualitative element | Key themes included  Control of eating (subthemes: Reduced hunger, Reduced cravings for sweetness, Less frequent meals and snacks, Preference for whole foods, Easy to sustain)  Control of health (subthemes: empowered and increased  Positivity, Increased quality of life  Increased energy levels.)  Social eating (subthemes: Reduced enjoyment, Negativity from public/  Acquaintances)  Interactions with medical  Professionals (subthemes: Participant independence, Apathy of doctors, Conflict over some medications) | Study population weighted towards those who had achieved partial or complete remission. |
| 35 | Yancy (2020). | USA | Noninferiority Randomized Clinical Trial | N=263 outpatients, uncontrolled HbA1c, and body mass  index (BMI; calculated as weight in kilograms divided by height in meters squared) of 27 or  higher | Participants randomized to the Group Medical Visits (GMV) group (n = 136) received counselling  about diabetes-related topics with medication optimization every 4 weeks for 16 weeks, then every 8 weeks (9 visits).  Participants randomized to the Weight management/Group Medical Visits (WM/GMV) (n = 127) received  low-carbohydrate diet counselling with baseline medication reduction and subsequent  medication optimization every 2 weeks for 16 weeks followed by an abbreviated GMV  intervention every 8 weeks (13 visits). The WM/GMV intervention  included low-carbohydrate nutrition, physical activity,  and weight management counselling. Initially, carbohydrate intake was restricted to approximately  20 to 30 g per day with no specified caloric restriction. | Problem Areas in Diabetes (PAID) | PAID score was lower in the group that incorporated weight management through a low carbohydrate diet (WM/GMV) when compared to the GMV alone group at each follow up. At 16 weeks 17.5 vs 23.1. At 32 weeks 17.4 vs 22.5 and at 48 weeks 18.3 vs 20.3. | Diabetes remission,  12 of 109 WM/GMV participants (11.0%) and no GMV  participants achieved an HbA1c level <6.5% after receiving  metformin alone or no diabetes medication. So differences in PS factors can only be interpreted as linked to the intervention not remission. |
| 36 | Zou et al (2022) | China | Single arm | N=125 <20 years after T2DM diagnosis, ≥6 months treatment with oral anti-diabetic drugs, without serious complications, and no history of insulin use | Participants divided into 2 groups: the obesity group (BMI ≥25 kg/m2) and the lean group (BMI <25 kg/m2). Both groups withdrawal of anti-diabetic drugs and alcohol, a low-carbohydrate diet. 1,800 kcal/day for the lean group, and 700–900 kcal/day for the obese group. Resistance and aerobic exercise. Restriction followed by a stepped normal construction diet. | None | N/A | Authors note that verbally participants discussed improved Q of L, reduced depression etc but this was not measured. |
