# Supplementary material for: Psycho-social factors associated with type two diabetes remission through lifestyle intervention: A scoping review
Source: PLoS One. 2023 Nov 16;18(11):e0294344. doi: 10.1371/journal.pone.0294344 (PMC10653481; doi:10.1371/journal.pone.0294344)
Supplement: S5 Table — (DOCX) [file pone.0294344.s005.docx]

**S5 table: Data extraction form two- detailing author and related studies, psycho-social interventions and cited studies which underpin the interventions.**

| Number | Author and related studies | Psycho-social intervention | Cited studies which underpin | Notes |
| --- | --- | --- | --- | --- |
| 1 | Ades et al 2015 | Termed a ‘behavioural weight loss program’. 24 weekly group ‘counselling’ sessions-focused on self- monitoring and action planning. Diet/exercise based individual sessions. | References Brownell (2004) LEARN programme for weight management | LEARN includes:” (a) self-monitoring of eating behavior; (b) controlling eating stimuli (c) physical activity; (d) nutrition education; (e) modifying self-defeating thoughts and negative emotions associated with dieting and body image; (f) setting realistic goals; (g) relationships; and (h) relapse prevention and weight maintenance.”(Carels et al., 2011) |
| 2 | Athinarayan et al 2019 | Nutritional information and biomarker tracking through an app. ‘education and support regarding dietary changes, behaviour modification techniques for maintenance of lifestyle changes’ Access to online peer support community | References Hallberg et al (2018) |  |
| 3 | Bhatt et al 2017 | Home blood glucose monitoring. Weekly remote consultation with nutritionist for ‘compliance’ | N/A | Monitoring for ‘compliance’ not support |
| 4 | Bynoe et al 2019 | “Intervention was delivered by a family practitioner (KB),  following a detailed study protocol that included how to  provide support to participants to address challenges in  adhering to the dietary guidance. Participants were encouraged  to phone KB between visits should they need advice.” | N/A | No access to protocol so unable to extract any further information |
| 5 | Correa et al 2022 | Use of CGM- biofeedback | N/A |  |
| 6 | Cox et al 2019 | Health coaching and educational sessions | N/A |  |
| 7 | Dave et al 2019 | Dietary counselling, exercise counselling, trained to self-monitor blood sugar. Care and support from one designated case  manager who was a qualified and experienced diabetes educator (DE). Carer education and support. Each counselling  session at the clinic was planned on a one-to-one basis and though  recommended on a quarterly basis, could be planned as required. | N/A | Unclear nature of the ‘counselling’ |
| 8 | De Hoogh et al 2021 | Personalised treatment plan linked to diabetes  subtype, other clinical parameters, current lifestyle behaviour, and personal preferences: dietary, physical activity, sleep, stress, and medication related advice. Shared decision making between multidisciplinary team. ‘Regular’ contact between the patient, nurse practitioner, and/or dietician in face-to-face consults, via e-mail, and via telephone. Subjects were supplied with a glucose meter to monitor the effect of their lifestyle adaptation on glucose levels. | N/A | Unable to access protocol |
| 9 | Esposito et al 2014 | Dietary/exercise advice. | N/A | Adherence |
| 10 | Gregg et al 2012 | Behavior Modification Curriculum: The 6 month, 16-session DPP protocol was modified for Look AHEAD to include group treatment and the changes in diet and activity. Each group session typically introduces one or two new topics in behavioural weight control, including recording food intake and physical activity, eating at regular times, limiting times and places of eating, and coping with negative thoughts related to overeating. All major topics are accompanied by a homework assignment. Of these, recording daily food intake and physical activity are likely the most important. Participants total their calories for each day (and for the week) and report at group sessions their success in meeting their target.  7-12 month: The curriculum for months 7−12 distinguishes between behaviors required to lose weight and those associated with maintaining a weight loss. Learning to reverse small weight gains, as they occur, is a critical skill for maintaining a weight loss. Sustaining motivation for behavior change is another key focus of this stage, given the decreased rewards (principally weight loss) of treatment. To this end, a motivational campaign which focuses on increasing physical activity is introduced early after the transition to bi-monthly group sessions. Other new concepts presented in months 7−12 include coping with dietary lapses, improving body image and self esteem, and expanding exercise options.  Monthly in-person sessions focus on individualized assessment of progress, review of self-monitoring records, problem solving of difficulties encountered, and goal setting. Sessions reinforce behavioral strategies introduced in the first year and, when appropriate, apply them to the problems of lapse and relapse | References: Wadden et al. (2004), Wadden et al. (2005), Baker & Kirschenbaum (1998), O’Neil & Brown (2005), Wadden et al. (1994), Rothman (2000) and Jeffery et al (2000). | Information from Look AHEAD research group: The Look AHEAD Study: A Description of the Lifestyle Intervention and the Evidence Supporting It (not original paper) |
| 11 | Lagger et al 2018 | 5-day outpatient Therapeutic Education Program (TPE) program-10 to 12 group session as well as about 8 to 10 individual sessions.  Followed by 12 individual monthly sessions lasting 1-1.5 h,  T.P.E had 5 dimensions :  – cognitive: patients received information in the form of  explanations or metaphors and constructed or criticized  their models of thought;  – affective: patients expressed their emotions, could feel the  unconditional support to approach sensitive subjects;  – perceptive: patients worked on their body sensations,  feelings, experienced the effect of a certain behavior or  treatment;  – infra-cognitive: patients were challenged by the confrontation  of contradictory information, interrogation of the  intimate reasonings, the foundations of thought;  -meta-cognitive: patients were led to analyze their purpose,  their sense of learning, their motivation for change. | References: Golay et al. (2009) |  |
| 12 | Lean et al ( 2018), Lean et al (2019), Rechakova et al, (2021) and Thom et al (2021) all focused on DiRECT | An ongoing structured  programme with monthly visits for long-term weight loss  maintenance. Physical activity and sleep were objectively  measured over 7 days by use of wrist-worn triaxial  accelerometers. For the  maintenance phase up to 24 months participants were  offered monthly 30 minute appointments with the dietitian or practice nurse, using tailored workbooks. |  | References Leslie et al (2016) DiRECT protocol but does not give details of psycho-social interventions |
| 13 | Marples et al 2022 | Use of readiness to change questionnaire. Initial phase participants were supported in groups by the trained HDC MDT. Sessions group-based face-to-face setting/ Covid= video consultation. 12-week TDR phase,  participants attended seven structured group sessions (bi-weekly). Content included goal setting, planning and side-effects and relapse-management, which focused on behaviour change techniques to facilitate long-term change. Food re-introduction phase: five group sessions (bi-weekly). Content included behavioural strategies, nutrition education, the encouragement of a structured meal pattern and lifestyle planning. Weight loss/rescue plan: Sessions focused on relapse management, nutrition education, managing difficult circumstances (e.g., eating out) and behavioural strategies. Participants were offered up to five one-to-one psychological support sessions (45 min per session) provided by the psychological therapist. |  | References Leslie et al (2016) DiRECT protocol but that does not give details of psycho-social intervention- unable to access supplementary information |
| 14 | Mottalib et al 2015 | Use of blood glucose monitoring. Structured modified dietary intervention. Individualized balanced and graded exercise plan.  Cognitive behavioural support: Group behavioural support sessions led by a clinical psychologist were conducted weekly during the initial 12 weeks then once monthly during follow-up. The sessions incorporated key components of cognitive-behavioural therapy for weight loss already validated in other clinical trials. These components included self-monitoring of eating and exercise, behavioural goal setting, stimulus control techniques, cognitive restructuring, assertive communication skills, stress management, and relapse-prevention. The monthly support group discussion was focused on active problem solving for relapse prevention and weight loss maintenance.  Group education Group didactic sessions were conducted each week for 30 minutes by a diabetologist, an exercise physiologist, a registered dietitian, or psychologist during the initial 12 weeks. | References  Knowler et al. (2002) Wadden & Osei. (2002) | Protocol of Why WAIT (Hamdy & Carver,2008) |
| 15 | Oser et al 2022 | Use of CGM- biofeedback. Empowerment program based on biological and self-reported feedback. Self-guide educational booklet. | References Anderson et al. (2002) and Hernandez-Tejada et al. (2012) |  |
| 16 | Rechakova et al 2017, Rechakova,et al 2019, Steven et al 2016 (Counterbalance study) | One-to-one support was provided weekly by telephone, e-mail, text message, or face-to-face contact. During the 6-month weight maintenance phase, participants were supported by a structured individualized program based on goal setting, action planning, and barrier identification, with monthly reviews. | References Michie et al. (2011) | Emphasis on adherence |
| 17 | Rein et al 2022 | CGM- biofeedback | N/A |  |
| 18 | Reid-Larsen et al 2019 | Self-monitoring of behaviours, perceived stress level, mood and motivation- linked to self-regulation theory  Diabetes management education and networking including group based counselling and online peer support forum | References Kanfer (1991), Burke et al. (2011) Deakin et al. (2005), Trento et al. (2001) and George et al. (2013) | Information from protocol not original paper |
| 19 | Romana et al 2019 | None | N/A |  |
| 20 | Sarathi et al 2017 | None | N/A |  |
| 21 | Taheri et al 2020 | **Self monitoring**• Identifying the relationship between mood and food selection and intake this will be accomplished using a food and mood diary• Using scales to gauge hunger and satiety• Regular weight measurement  • Recording of food selection and intake **Goal setting**• SMART goals will be used throughout• Individualised goals will be set by participants regarding diet and physical activity  **Stimulus control**• Identification of external and internal triggers to unhealthy eating habits • Use of distraction techniques to help subjects avoid emotional eating  **Cognitive restructuring** • Reviewing the impact of negative thoughts and beliefs and their impact on behaviour change.  • Replacing negative thoughts and beliefs with positive thoughts and beliefs  **Eating behaviour**• Education about healthy eating behaviours  • Choosing low energy alternatives to high energy foods  • Portion control  • Timing and duration of eating  • Dealing with snacking  **Planning**• Planning meals and physical activity  • Planning for challenging situations e.g. eating with family, eating at restaurants, and eating and activity while traveling  Maintenance • Maintaining successful behaviours  • Dealing with lapses and avoiding relapse  • Avoiding previous behaviours that resulted in weight regain  **Reward & support**• Non-food related rewards  • Support from family and friends  **Problem solving**• Problem solving skills  • Dealing with high risk situations that impact on eating | Protocol: references  Butryn et al. (2011) | Information from protocol not original paper |
| 22 | Umphonsathien et al 2019 | Blood glucose checking | N/A | Can’t access protocol |
| 23 | Umphonsathien et al 2022 | Blood glucose checking | N/A | Can’t access protocol |
| 24 | Unwin et al 2020 | For patients who opted to try a lower carbohydrate diet,  dietary advice was given as part of routine GP or practice  nurse consultations. The level of ongoing support  was tailored to patient choice and clinical need. In addition  to 10 min ‘one-to-one’ appointments (we estimate  an average of 3 appointments per patient, per year were  required), the practice offered access to optional 90 min  evening group sessions that ran approximately once every  6 weeks. Group sessions included a psychologist who facilitated  behaviour change by encouraging participants to  consider their individual health goals, the resources available  to them, setting realistic steps and enabling the individual  to notice what works for them. Patient relatives  and carers were encouraged to attend as some patients  relied on others for food shopping or cooking. Group  sessions also provided a forum for patients to offer practical  support to their peers and for the training of new  staff. | References: Unwin (2019) GRIN model |  |
| 25 | Webster et al 2019 | None | N/A |  |
| 26 | Yancy et al 2019 | Participants in the WM/GMV groups met every 2weeks for  16weeks for WM counseling and medication management and  every 8weeks thereafter for diabetes counselling, medication  management, and continued WM support. Classes covered topics, such as grocery shopping, restaurant eating, and recipe makeovers, and  incorporated behavioural techniques to improve adherence.  From protocol: self-monitoring encouraged. Partners/family encouraged to attend group sessions. | N/A | No indication of what ‘behavioural techniques’ were incorporated. |
| 27 | Zou et al 2022 | Ongoing support and encouragement was provided by strengthened management, which comprised frequent consultations, including a 30-minute weekly face-to-face interview, 3×10-minute daily consultations via WeChat or regular telephone calls. | N/A | Lack of clarity on nature of ‘consultations’ |
